# Holistic Approach toward a Damage-Less Sputtered Indium Tin Oxide Barrier Layer for High-Stability Inverted Perovskite Solar Cells and Modules

**DOI:** 10.1021/acsami.2c10251

**Published:** 2022-11-02

**Authors:** Sathy Harshavardhan Reddy, Francesco Di Giacomo, Fabio Matteocci, Luigi Angelo Castriotta, Aldo Di Carlo

**Affiliations:** † Centre for Hybrid and Organic Solar Energy (CHOSE), University of Rome Tor Vergata, Rome 00133, Italy; ‡ ISM−CNR, Institute of Structure of Matter, National Research Council, Rome 00133, Italy

**Keywords:** stability, perovskite solar cells, low-temperature processing, perovskite solar modules, ITO, sputtering damage

## Abstract

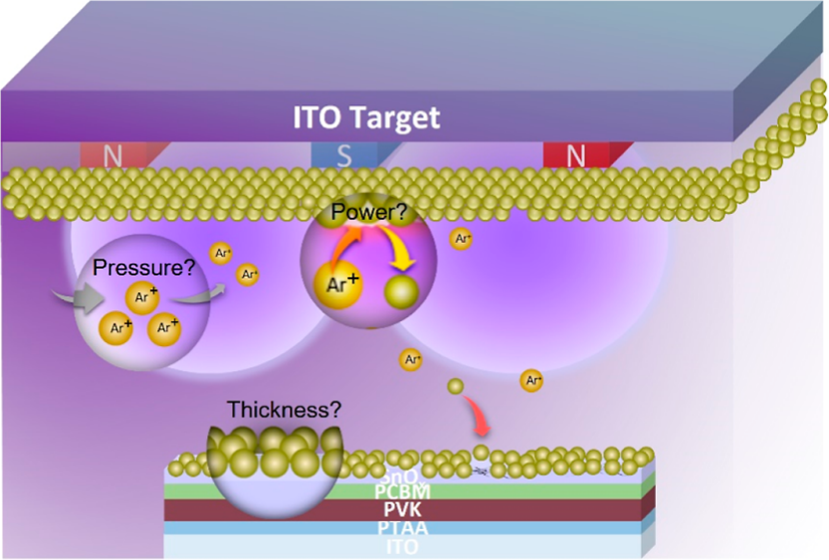

The commercialization of perovskite solar cells (PSCs) requires the development of long-term, highly operational-stable devices. An efficient barrier layer plays a key role in improving the device stability of planar PSCs. Here, we focus on the use of sputtered indium tin oxide (ITO) as a barrier layer to stop major degradations. To mitigate efficiency losses of cells with the ITO barrier, we optimized various sputtering process parameters such as ITO layer thickness, target power density, and working pressure. The fabricated planar inverted PSCs based on the novel ITO barrier optimization demonstrate a power conversion efficiency (PCE) of 19.05% on a cell area of 0.09 cm^2^. The encapsulated cells retained >80% of their initial efficiency after 1400 h of continuous illumination at 55 °C and 94.5% of their initial PCE after 1500 h stored in air. Employing such a holistic stabilization approach, the PSC minimodules without encapsulation achieved an efficiency of 16.4% with a designated area of 2.28 cm^2^ and retained approximately 80% of the initial performance after thermal stress at 85 °C for 350 h under ambient conditions.

## 1 Introduction

With the great efforts of the photovoltaic community, hybrid organic–inorganic perovskite solar cells (PSCs) have reached unprecedented power conversion efficiencies (PCEs) as high as 25.7%,^1^ making them the most promising next-generation solar cells. Albeit the impressive PCEs, stability remains a major challenge that decides the future of commercialization. Researchers across the globe are striving for novel ways to suppress the degradation and improve the stability of PSCs. The major degradation in PSCs results from the complex photo- and electrochemical processes under UV, moisture, light, temperature, and electrical bias.^2−6^ Widely known degradations in PSCs stem from ingression of moisture,^7−9^ egression of volatile species,^10,11^ metal migration,^12^ and corrosion of metal electrodes by interaction with halides in the perovskite.^13^ To improve the stability of perovskite devices, several methods have emerged,^14^ including the investigation of novel charge transport and electrode materials for improved stability, as well as engineering perovskite layer composition and fabrication strategies.^15^ Several strategies have also been investigated to improve the stability of devices, with *T*_80_ of thousands of hours being proven under various moisture, temperature, and illumination conditions. However, all strategies are aimed at addressing one type of degradation. The quest to find a holistic solution to prevent all the degradation pathways still remains open.

Sputtering transparent conductive oxides, such as indium tin oxide (ITO),^16,17^ zinc-doped tin oxide (ZTO),^16^ aluminum-doped zinc oxide (AZO),^18,19^ or indium zinc oxide (IZO),^20^ as a top contact, has emerged as a promising approach that addresses most of the degradation processes. ITO evolved as one of the best solutions that address the majority of the instability issues. Having good diffusion barrier properties, ITO can stop the effusion of volatile organic species and prevent the migration of ions and metal atoms as well. Moreover, ITO has high electrical conductivity^21^ and optical transmittance^22^ in the visible and near-infrared region, making it an excellent electrode for applications in semitransparent and tandem solar cells. Furthermore, a metal electrode on top of ITO could be used as a back-reflector to generate more current in the device. Previously many reports have shown to employ sputtered ITO as a top electrode to increase the overall stability of the device.^17,23^^,^ However, the PCEs of ITO-sputtered devices are relatively lower when compared to their reference devices.^24−26^ In fact, few articles demonstrate the incorporation of ITO for stability but shy away from showing the PCEs.^27^ According to the most likely explanation, sputter methods might harm the charge-transport layers and the underlying perovskite layer, limiting device performance.^28,29^ Sputter damage can affect either the underlying charge-transport layers or the absorber layers, wherein structural changes, such as the dissociation of chemical bonds, can hamper the performance.^30^ It can be noted that the energies of sputter atoms (10 eV), negative Ar ions (5–15 eV), negative ions formed at the target (110 eV) (depends on target voltage), positive ions formed in the plasma (15 eV), reflected atoms and neutralized ions from the target surface (17–25 eV), and UV light (3.2–9.9 eV) are sufficiently high to damage the layers, especially the organic ones.^31,32^ A more detailed discussion on the sputtering damage can be found elsewhere.^33^ To overcome the sputter damage to the underlying layers, many approaches have been put forward, such as inserting appropriate buffer layers,^34^ lowering the power densities,^35,36^ changing the sputter gas,^37^ and intermittent sputtering deposition.^38^ In n–i–p architecture, Park et al. demonstrated a surface modification of tungsten oxide with niobium oxide, increasing the fill factor (FF) from 70.4 to 80.3%; however, the *V*_oc_ of the device was reduced.^39^ Although the static sputtering outside the target area dramatically reduces the sputtering damage, the wastage of the material and low throughput are its drawbacks. Bett et al. demonstrated a low power density strategy to show reduced sputter damage; however, the authors did not perform an in-depth investigation to determine the range of power densities that can withstand the damage.^36^ To mitigate the sputtering damage, Zhao et al. reported a sequential deposition of thin layers of Ag (1 nm) and MoO_x_ (3 nm) through thermal evaporation.^34^ The presence of a thin metal can induce thermal degradation and further high-vacuum techniques also yield low throughput. Despite the use of buffer layers, the damage is evident, reducing the performance when compared to their reference devices. Methods such as the intermittent sputtering deposition mitigate the thermal damage during sputtering but have a huge waste of expensive ITO as the shutter is open only for 1 s for every 7 s. Therefore the proposed solutions still have several shortcomings. Moreover, an in-depth investigation into the optimization of the sputtering process is missing in the literature.

In this work, ITO is introduced as a barrier layer to improve the overall stability of the PSC. Thin films of ITO are deposited via an RF magnetron sputtering by varying the experimental setup parameters during the deposition. First, we investigated the role of thickness followed by the target power density and working pressure of the chamber. We used a solution-processed SnO_x_ buffer layer to protect the device from sputtering damage. The solution-processed SnO_x_ contains organic dispersants that stabilize the nanoparticle dispersion, and these components may induce a faster degradation, particularly with the copper metal (see Figure S1). Despite the faster degradation, the use of SnO_x_ is critical to our experiments since it protects the cell from sputtering damage. Furthermore, this work intends to optimize the sputtering process of ITO as a barrier layer without scarifying the PCE and stability. The goal of this study is to demonstrate the feasibility of manufacturing the ITO barrier layer on most p–i–n architectures to improve the stability tremendously while retaining the PCE of the PSCs and modules. In addition, this study has wider implications for single-junction, semitransparent, and tandem devices.

## 2 Results and Discussion

We fabricated p–i–n planar PSCs on an ITO-coated glass substrate. Our stack is based on a triple cation perovskite layer with nominal composition Cs_0.05_FA_0.81_MA_0.14_PbI_2.7_Br_0.3_ along with BMIM-BF_4_ ionic liquid additives, labeled as 3C-IL. The device architecture comprises glass/ITO/PTAA/3C-IL/PCBM/BCP/Cu. All the fabrication details can be found in the Experimental Section. Initially, a 100 nm-thick ITO layer was sputtered on top of BCP using a shadow mask defining four 5.5 mm × 5.5 mm areas on each substrate. This was followed by the evaporation of Cu with a shadow mask defining four 3.5 mm × 3.5 mm pixels aligning with ITO. This was intentionally done to subside any migration of copper atoms through the sides. These devices showed decreased PCEs owing to the damage induced by sputtering.^40,41^ The BCP/ITO/Cu device showed an open-circuit voltage (*V*_oc_) of 1.05 ± 0.07 V, a short circuit current density (*J*_sc_) of 17.5 ± 0.8 mA/cm^2^, and a FF of 52.6 ± 6.4%, which yields a PCE of 9.6 ± 0.9%. The low FFs in the devices can be due to the sputtering damage as reported previously.^40^ All the photovoltaic characteristics of the ITO-based devices reported in this article are usually measured after 1–2 weeks of fabrication unless specified otherwise. It is worth noting that the ITO-based devices typically increase their PCEs over 1–2 weeks due to the significant increase in the FFs. We speculate that this reversible degradation might be due to the slow self-healing nature of the device after being removed from the harsh UV–plasma environment of the sputter (see Figure S2).^20,42^

**Figure 1:**
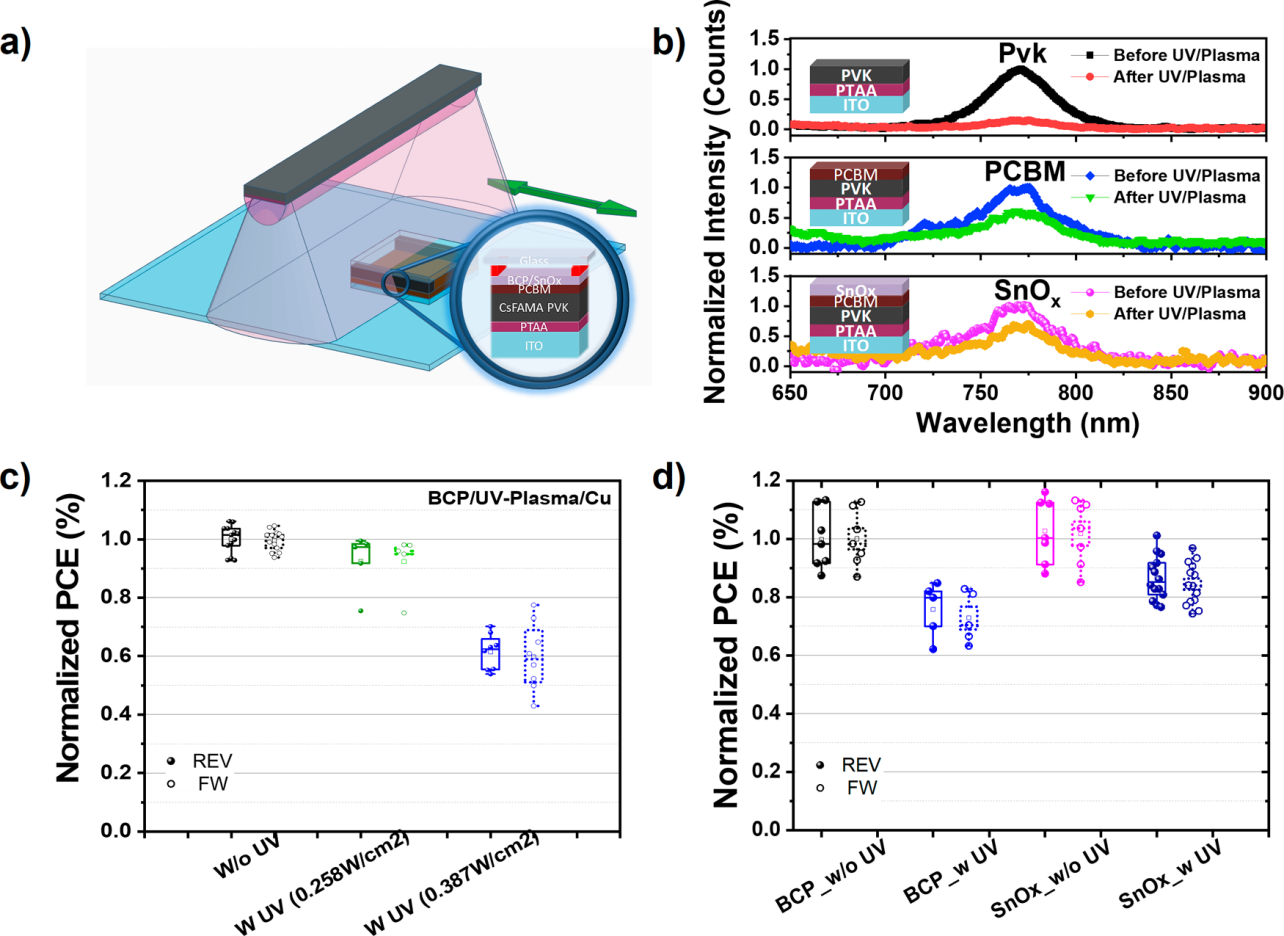
Impact of sputter damage. (a) Schematic diagram of the experimental setup to decouple the UV/plasma sputtering damage from hard bombardment damage of particles. (b) PL of the stacks fabricated to the perovskite (Pvk), PCBM, and SnO_x_, before and after the UV/plasma treatment upon irradiation at a power density of 0.516 W cm^–2^ and a rate of 1.86 nm/min. (c) Performance statistics of ITO/PTAA/3C-IL/PCBM/BCP/Cu devices with and without UV/plasma at different sputtering power densities. The solid-filled symbol and open symbol indicate the *J–V* parameters measured under the reverse-bias (REV) and forward-bias (FW) scan directions, respectively. (d) Comparison of the statistically normalized PCE of PSCs with BCP and SnO_x_ buffer layers. The solid-filled symbol and open symbol indicate the *J–V* parameters measured under the REV and FW scan directions, respectively.

In general, the damage due to the sputtering can be from the bombardment of high-kinetic-energy plasma particles and sputtered particles on the substrate or due to the coupled effect of UV/plasma light.^43,44^ According to the literature, it is unclear as to what the prominent source of damage is. To contribute to a better understanding of this, the emission spectrum of the sputter was measured through the window of the sputtering chamber (see Figure S3). The spectrum reveals that the peaks at the wavelength (λ) 325 nm correspond to UV, while the peaks at 450 and 600 nm may correspond to the plasma.^45,46^ To decouple the bombardment damage from UV/plasma damage, we performed an experiment in which the ITO/PTAA/3C-IL/PC_61_BM/BCP device was masked with a glass microscope slide and subjected to UV/plasma (see Figure 1a). This enables only UV/plasma treatment of the device, rather than the harsh bombardment of plasma particles. After the treatment, the device was deposited with a 100 nm layer of copper (Cu). Upon measuring the *J–V* characteristics, the UV/plasma-treated devices yielded lower PCEs, while the untreated devices showed higher PCEs. The performance characteristics are illustrated in Figure 1c. Upon increasing the sputtering power density from 0.258 to 0.387 W cm^–2^, the damage appears proportional. This experiment clearly demonstrates the damage induced by the UV/plasma radiation in the sputtering process. Our results are in agreement with a similar study in crystalline silicon devices.^43^ To evaluate if the damage due to UV/plasma persists upon replacing the BCP layer with the SnO_x_ buffer layer, we performed the same experiment. As shown in the statistical data summarized in Figure 1d, the SnO_x_ buffer layer devices also showed a decrease in their performance when irradiated with UV/plasma. However, the reduction of the PCEs in SnO_x_-based devices is relatively less than in the BCP-based devices. These results can be attributed to the slightly better damage tolerance of the SnO_x_ layer. In summary, the SnO_x_ buffer layer cannot fully protect from sputtering damage. To determine which layer is affected by the UV/plasma, the same experiment has been conducted with substrates fabricated with layers up to the perovskite, PCBM, and SnO_x_. The photoluminescence (PL) spectrum was measured for all the samples before and after the UV/plasma treatment. As shown in Figure 1b, all the samples treated with UV/plasma showed quenched PL spectra. Although SnO_x_-based samples demonstrated relatively lower quenching than samples with PCBM and perovskite layers, they still suffer from the damage caused by sputtering. This demonstrates that the UV/plasma indeed damages all layers at the time of sputtering despite the use of buffer layers. Furthermore, we speculate that the perovskite layer might be damaged by UV/plasma, resulting in a lower performance of the devices. Thus, we can preliminarily infer that apart from the damage caused by the fast bombardment of atoms, UV/plasma also induces significant damage despite the use of buffer layers.

**Figure 2:**
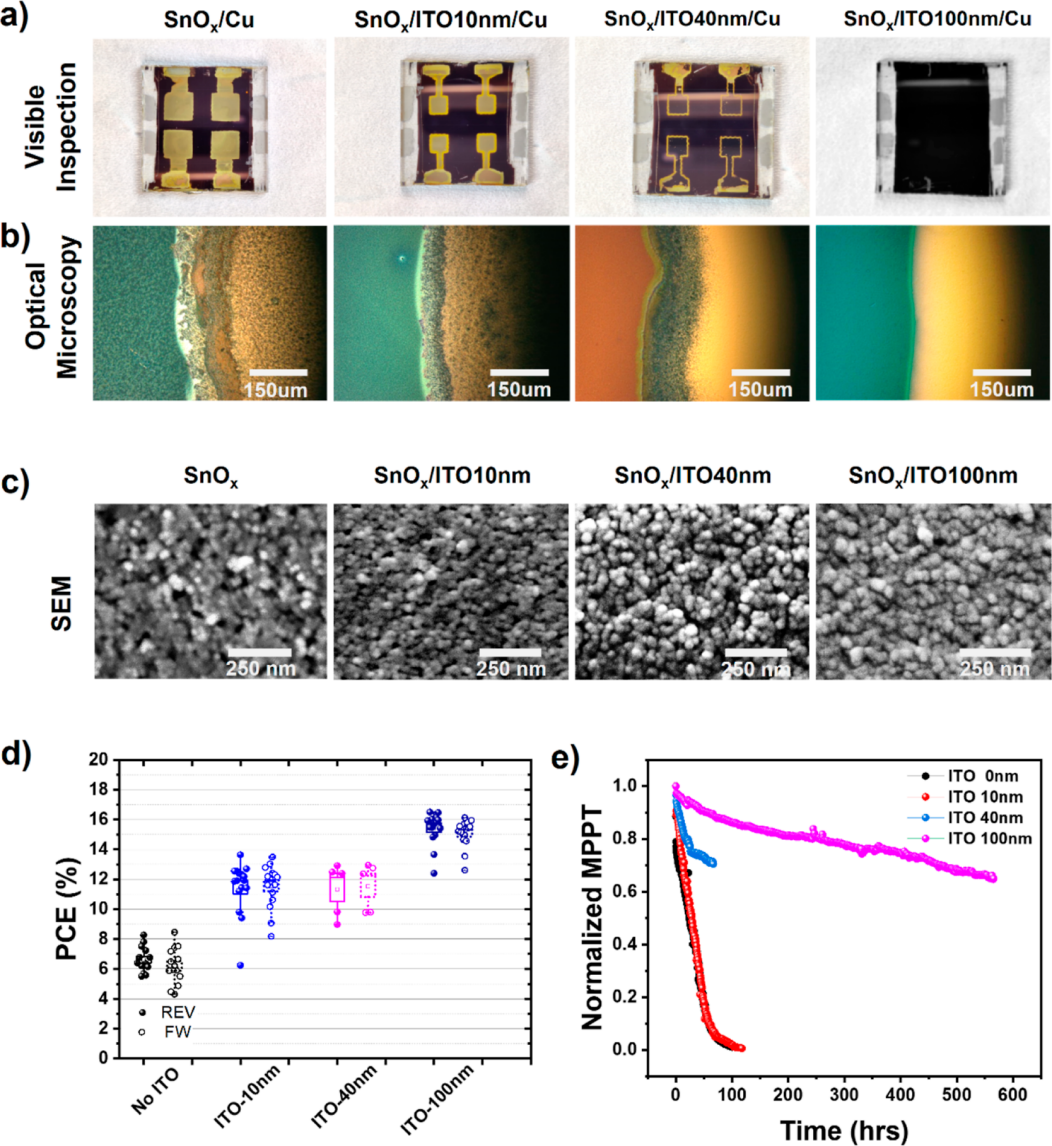
Morphological characterization of the PSC without and with 10, 40, and 100 nm ITO incorporation by (a) visible inspection through camera (top row), (b) optical microscopy (middle row), (c) SEM images on samples without Cu on top (bottom row). (d) PCE distribution of aged PSCs without and with the incorporation of 10, 40, and 100 nm ITO. The solid-filled symbol and open symbol indicate the *J–V* parameters measured under the REV and FW scan directions, respectively. (e) Light-soaking stability of devices with different ITO thicknesses monitored at 55 °C under continuous light soaking (white LED, with a light intensity to simulate 0.7 sun AM1.5G solar irradiation).

The thickness of ITO in a semitransparent solar cell is usually optimized by maximizing its optical transparency and electrical conductivity. However, in the devices where ITO is used as a buffer layer between the transport layer and metal electrode, the optimization is not related to the optical transparency. Few studies in the literature reported the stability of ITO-based devices but did not quantify the thickness employed.^47,48^ However, some studies reported thicknesses of 40,^49^ 150,^16,23^ 180,^50^ and 270 nm^51^ without specifying thickness optimization. Undoubtedly, depositing a large amount of ITO on top of the device can improve stability. However, it will only increase the processing time and utilize more indium. Therefore, to close this gap, we performed a thorough investigation to determine the optimal thickness of the ITO buffer layer. Moreover, the thickness optimization of the ITO layer would facilitate the improvement of the stability of the PSCs without reduction in the PCE. The thickness was varied by tuning the number of scans of the pallet according to the deposition rate that was measured for each set of deposition for different power densities of the target. In a single experiment, the devices were tested at three different thicknesses of ITO (10, 40, and 100 nm). The ITO film thickness in PSCs has a significant influence on the morphology and electrical properties in the bulk and at interfaces. At 10 nm, the sheet resistance is around 450 Ω/sq: upon increasing the thickness to 40 and 100 nm, the sheet resistance decreases to 90 and 30 Ω/sq, respectively. When the devices were encapsulated with Kapton tape and stored in air for over 1000 h, physical degradation was observed (see Figure 2a). Optical microscopy images reveal that when there is no ITO, copper interacts with the perovskite (and possibly with organic dispersants present in the solution-processed SnO_x_ layer), causing the device to degrade. On increasing the thickness of ITO, the degradation of the device is reduced. Visual examination of the 100 nm-thick ITO showed no signs of degradation. Figure 2b shows the optical microscopy images of the ITO/Cu interface, suggesting that 100 nm is sufficient enough to stop such degradation. As observed from the scanning electron microscopy (SEM) images in Figure 2c, the growth of ITO replicates the morphology of the SnO_x_ nanoparticle layer. The morphology of the SnO_x_ layer contains small pinholes which might allow copper migration to the perovskite. The presence of such tiny pinholes can also be seen on 10 and 40 nm ITO layers. Increasing the ITO thickness from 10 to 40 nm such as pinholes was minimized. Moreover, the degradation starts at the edges of ITO–copper interfaces, likely because the edge thickness of the ITO/Cu interface is smaller than the center thickness owing to the sputtering mask’s shadow effect. Interestingly the SEM image of 100 nm ITO shows a compact morphology without any pinholes. Such a layer with all the pores covered showed no signs of degradation. The *J–V* characteristics of the devices (stored for 2 weeks) are shown in Figure 2d. A summary of the photovoltaic parameters of the corresponding devices is shown in Table S1 (measured immediately) and Table S2 (measured after storage for 2 weeks). Furthermore, we investigated the long-term stability of the ITO-based PSCs at different thicknesses in an ambient atmosphere with a humidity of ca. 45% under a light soaker with a 0.7 sun illumination (Figure 2e). The reference, 10, and 40 nm ITO-based devices all showed a reduced stability of *T*_80_ < 50 h. However, the 100 nm device retained 80% of its initial efficiency after >400 h. Such high long-term stability of device performance can be attributed to the better diffusion barrier properties of the thick ITO film. Therefore, 100 nm-thick ITO is incorporated in all the remaining sputtering parameter optimizations.

**Figure 3:**
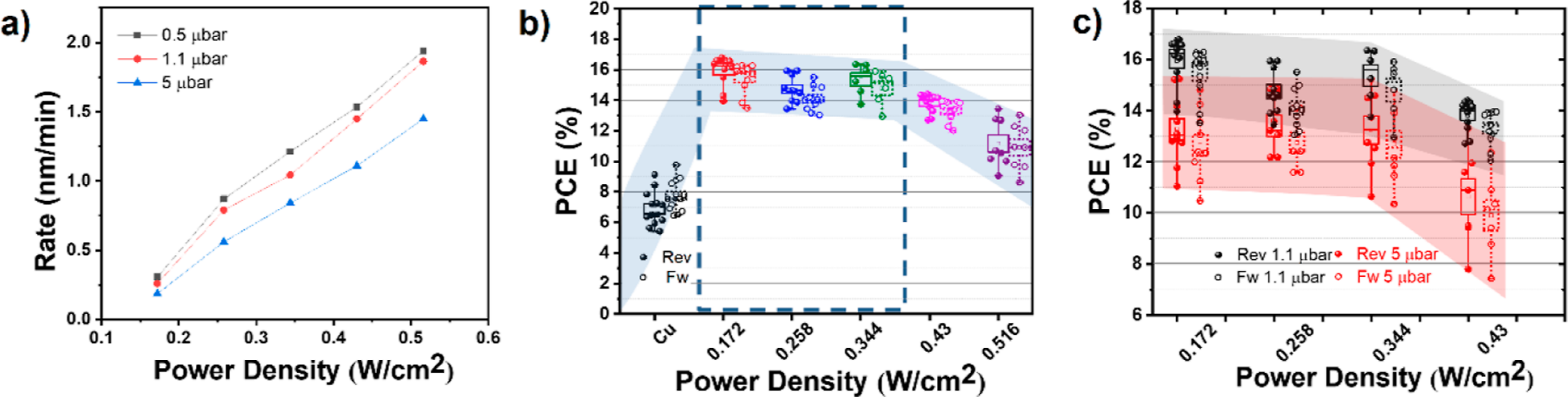
Optimization of sputtering parameters. (a) Deposition rates of ITO films according to the sputtering power density. (b) Performance of PSCs (glass/ITO/PTAA/Cs_0.05_MA_0.14_FA_0.81_PbI_2.7_Br_0.3_/PC_61_BM/SnO_X_/ITO/Cu) at various power densities. The solid-filled symbol and open symbol indicate the *J–V* parameters measured in the REV and FW scan directions, respectively. (c) Performance evolution of the respective PSCs under various working pressures. The solid-filled symbol and open symbol indicate the *J–V* parameters measured under the REV and FW scan directions, respectively.

It is essential to identify the target voltage required to minimize the impact of energetic particles causing damage. It has previously been demonstrated that decreasing the power density^52^ and raising the working pressure^53^ results in a drop in discharge voltage. However, raising the working pressure also means slower deposition and prolonged UV irradiation. The sputter process parameters such as target power density and working pressure in the chamber are the key elements for the optimization of deposition. In order to identify the sputtering parameters pertaining to 100 nm of ITO, we sputtered ITO by varying the number of scans of the pallet at a range of power densities and working pressures. The rate optimizations at different power densities were performed on the glass substrate. The rate of deposition versus power densities at various working pressures is illustrated in Figure 3a. It is observed that the slope of deposition rate at 0.5 and 5 μbar for all power densities seems comparable.

To illustrate the role of different sputtering parameters on the performance of PSCs, we sputtered ITO on top of the SnO_x_ layer by varying the RF power densities in the range 0.172–0.516 W cm^–2^ and maintaining the chamber pressure at 1.1 μbar at room temperature with an argon gas flow of 40 sccm. The sputter parameters were chosen in a way to reach a final layer thickness of 100 nm. The sheet resistance, transparency, and morphology measurements of the sputtered ITO at different power densities and working pressures seem comparable (see Figure S4). The photovoltaic parameters of the solar cells processed at different power densities are summarized in Table S3. The corresponding performance statistics are shown in Figure 3b. The *J*_sc_ and *V*_oc_ of these PSCs remain almost identical. However, the FF is significantly enhanced at low target power densities (0.172, 0.258, and 0.344 W cm^–2^) with respect to the ITO-free device. A further increase in the RF power density to 0.43 and 0.516 W cm^–2^ notably reduces the *J*_sc_ and FF, while the *V*_oc_ does not change much at a saturation level. In Table S3, the average series resistance of *R*_s_ has increased from 6.6 ± 0.6 to 11.5 ± 2.5 Ω cm^2^ with an increase of RF power densities from 0.172 to 0.516 W cm^–2^, which could be due to the intense UV/plasma sputtering damage. Increasing the RF power density increases the overall UV/plasma intensity, causing damage to the device irrespective of the presence of a buffer layer. The damage is evident from the increased series resistance and a subsequent reduction in both FF and PCE. We observed that the UV/plasma damage is lower in a range of RF power densities from 0.172 to 0.344 W cm^–2^. Despite the shunt resistance (*R*_p_) showing no clear trend of decrease or increase, the FF of the devices decreases from 70.6 ± 2.6 to 54.9 ± 5.7%, indicating the dominant contribution from *R*_s_. Furthermore, the devices perform the best in a range of target power densities from 0.172 to 0.344 W cm^–2^ with a deposition rate of 0.26 to 1.04 nm/min.

The chamber pressure value also impacts the deposition process. It is seen clearly in Figure 3a that the rate of the deposition at the 1.1 and 0.5 μbar does not change significantly. Therefore, the devices were tested at only two pressures (1.1 and 5 μbar). In Figure 3a, it can be seen that at a working pressure of 5 μbar, the UV power density is almost similar; however, the exposure time is doubled. In order to have an unbiased comparison of the results, we measured the current density versus voltage (*J–V*) curves of the devices after 1 week to further remove any kinds of reversible sputtering damage. Solar cells processed at 5 μbar have lower mean PCEs when compared to a working pressure of 1.1 μbar. As observed in Table S4, the overall PCE reduced due to the reduction of slight *J*_sc_, and a remarkable decrease in the FF was observed. It could indicate that at higher working pressures, the decreased PCEs might be due to the drastic increase in the UV/plasma exposure time. The interpretation of the *J–V* data is fairly challenging due to the large variation in the results. Interestingly, a pattern has been observed when comparing the *J–V* performance statistics at two different working pressures (see Figure 3c). At lower power densities, the spread-out is small and the PCE reduction is smaller. As the RF power density increases, the spread becomes larger, indicating that the time of exposure could also induce damage to the devices. In general, at high chamber pressures, more target particles are present in the chamber, causing the high-energy ions to decelerate and aiming at a gentler process. However, at such high working pressures, prolonged UV/plasma exposure could damage the underlying layers, leading to lower FFs. Moreover, at higher working pressures, the sheet resistance of ITO increased by 5–10 (see Figure S4a), which may not contribute to the decrease of the FF. From these results, it can be concluded that a working pressure of 1.1 μbar and a band of RF power densities ranging from 0.172 to 0.344 W cm^–2^ are beneficial for minimizing the sputtering damage.

**Figure 4:**
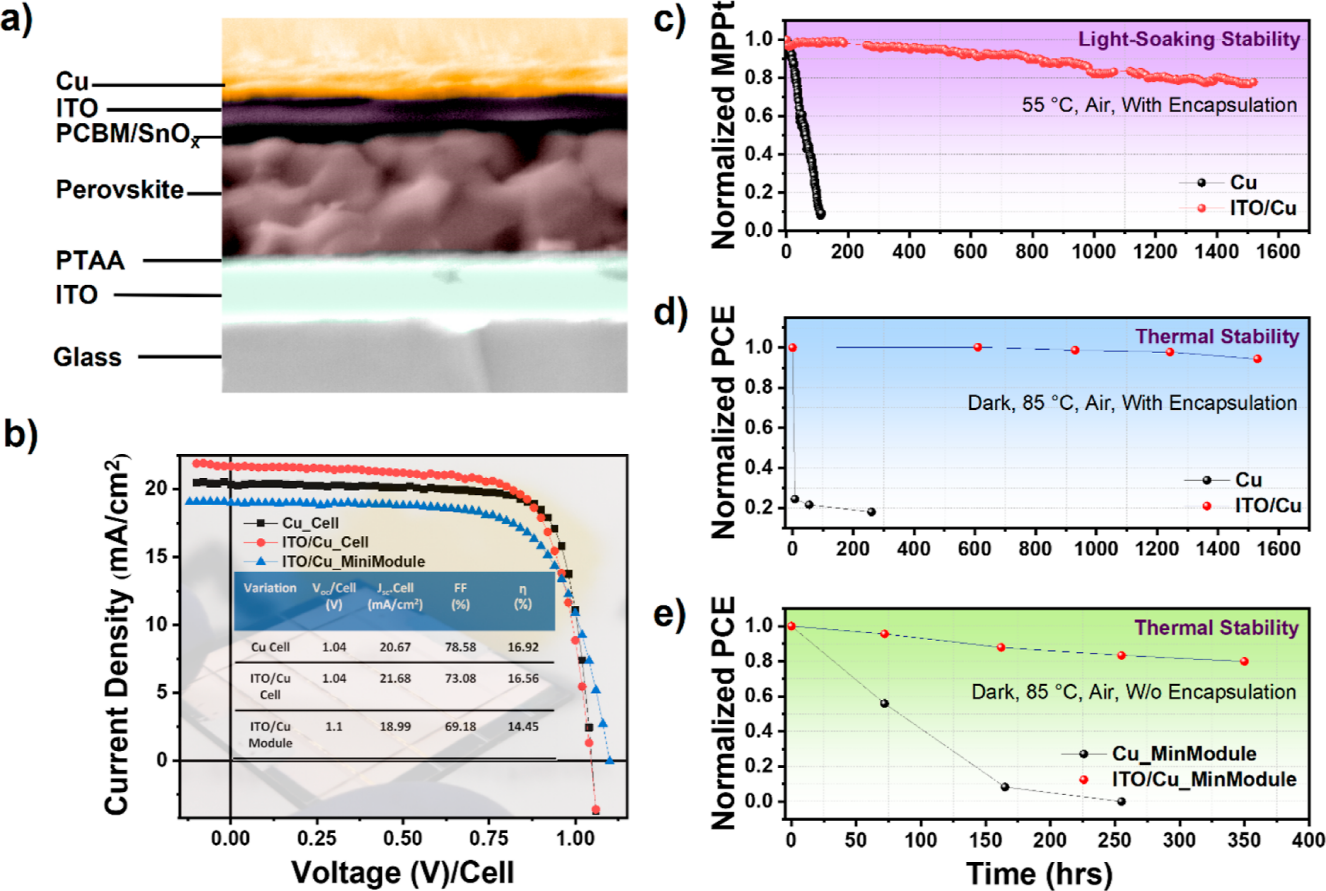
Performance evolution of ITO-based cells and modules. (a) Cross-sectional SEM image of the optimized ITO/Cu device. (b) The *J*–*V* curve depicts the parameters of best-performing champion devices. (c) Long-term stability of PSCs based on the ITO barrier layer, stored in inert conditions. The MPP tracking of the ITO-incorporated device was measured at a 0.7 sun illumination. (d) Thermal stability of corresponding PSCs with Kapton tape encapsulation in an ambient environment at 85 °C. (e) Thermal stability of corresponding minimodules without encapsulation in an ambient environment oven at 85 °C.

To evaluate the performance of solar cells featuring sputtered ITO films, we fabricated p–i–n PSCs and modules with the following structure: ITO(250 nm)/PTAA(18 nm)/3C-IL(460 nm)/PCBM(60 nm)/BCP/SnO_x_(40 nm)/ITO(100 nm)/Cu(100 nm), where the layer thicknesses have been measured by cross-sectional SEM. The thickness of the BCP layer is <5 nm, and it is undetectable in SEM (see Figure 4a). The addition of a thin BCP layer does not raise the PCE but rather increases the yields of fabrication and aids in the reduction of shunts (data not shown). The current–voltage (*J*–*V*) characteristics of PSCs with an area of 0.09 cm^2^ have been measured in a reverse-bias scan and the PCE of 16.56% for the cell with SnO_x_/ITO/Cu is comparable to that of 16.92% for the one with BCP/Cu (Figure 4b). The difference is mainly related to a slight decrease in the FF for the SnO_x_/ITO cell. The *J*_sc_ of devices with (w) SnO_x_/ITO is slightly better than that of devices without (w/o) SnO_x_/ITO. The incident photon-to-current efficiency (IPCE) spectra of w and w/o SnOx/ITO are shown in Figure S6a. It can be noted that the slight increase in the *J*_sc_ might be because of the enhanced back-reflection from ITO. We also demonstrate the light-soaking stability of PSCs encapsulated with Kapton tape kept in an air environment following the ISOS-L-1 light-stability protocol.^54^ The maximum power point tracking (MPPT) of the cell with ITO exhibited good light-soaking stability with a *T*_80_ of 1420 h, while the reference device without ITO exhibited a *T*_80_ of just 40 h (see Figure 4c). After the light-soaking measurement, immediately, the sample was measured at 1 sun under AM1.5 spectral conditions. The devices partially recovered to their efficiency (up to 85% of the initial efficiency). The 35-fold increment in the light-soaking stability is attributed to the robust ITO optimization. Thermal stability measurements were also performed on the Kapton-encapsulated devices. Following the protocols of ISOS-D-2, the devices were stored under 85 °C and ambient relative humidity in an oven and were taken out to measure the *J*–*V* curves under ambient conditions with a maximum-power-tracking program until the efficiency was stabilized. The reference device without ITO degraded quickly from 16.92 to 3.05% within 260 h (Figure 4d), while the ITO-based device demonstrated superior stability, degrading from 15.73 to 14.85% in 1530 h. The superior thermal stability can be due to the optimized 100 nm-thick ITO, preventing Cu from migrating into the device and providing a diffusion barrier to avoid sublimation of the organic component of the perovskite.

Similarly, minimodules with an area of 2.31 cm^2^ also demonstrated a decent PCE of 14.45%. The lower *J*_sc_ of the module when compared to the cell leads to slightly reduced efficiencies. This reduction in the *J*_sc_ arises from the lowest *J*_sc_ of the interconnected cells, as can be seen in the EL image in the Supporting Information, Figure S5c. Moreover, the slightly lower performance can also be attributed to the flawed P3 of ITO/Cu (see Figure S5d). It is also observed that a small pinhole in the perovskite or beneath layers could significantly impact by shunting the entire cell, obtaining lower *V*_oc._ The minimodules were unencapsulated and stored under 85 °C. Similar to the cells, the minimodule with ITO demonstrated superior thermal stability when compared to the minimodules without ITO. Figure 4e shows that the ITO-based minimodules demonstrate a *T*_80_ of 350 h, reducing the PCE from 12.64 to 10.1%, while the reference minimodule without ITO completely lost almost 100% of its initial PCE within 250 h. The ITO-based minimodules demonstrate lower thermal stabilities when compared to those of small-area PSCs (Figure 4e). The degradation mainly comprises a 23% loss in the FF, while losses in the *J*_sc_, and *V*_oc_ are insignificant. We believe that the relatively lower thermal stability, when compared to cells, can be due to the defects through which copper can migrate into the device, causing degradation. The copper deposited inside the P2 scribe can be migrated laterally into the device, thereby degrading the minimodules. Anyhow, we believe that the stability of P2 is enhanced by the deposition of ITO.

**Figure 5:**
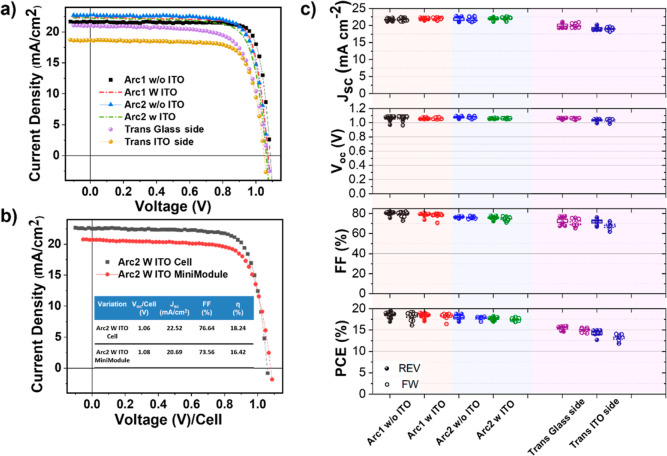
Performance comparison of high-performance PSCs with glass/ITO/HTL/PFN-Br/Cs_0.05_MA_0.14_FA_0.81_PbI_2.7_Br_0.3_-IL-OAm-BHC/PC_61_BM/SnO_x_/(ITO)/Cu. The label Arc1 indicates the use of MeO-2PACz as the HTL, and Arc2 and trans indicate the use of PTAA. The labels indicate if an interlayer of 100 nm of ITO is used before the metal contact. (a) Comparison of *J–V* curves of best-performing champion devices of various architectures with and without ITO. (b) Comparison of *J–V* curves of the best-performing ITO-based cell and module. (c) Performance statistics displaying a comparison of devices w and w/o ITO of various architectural devices. The solid-filled symbol and open symbol indicate the *J–V* parameters measured under the REV and FW scan directions, respectively.

The optimized ITO sputtering process was also tested on a more efficient structure to fully exploit its potential. A set of devices using the PFN-Br interlayer, MeO-2PACz HTL, and additional additives [oleylamine (OAm) and benzylhydrazine hydrochloride (BHC)] in perovskite are fabricated using the inverted solar cell architecture to obtain an improved solar cell performance.^15,47,55,56^ As shown in Figure 5, the device structure labeled Arc1 w/o ITO showed a PCE of 18.54 ± 0.69, while the corresponding ITO stack labeled Arc1 w ITO showed a comparable PCE of 18.44 ± 0.55%. Similarly, the device structure labeled Arc2 w/o ITO achieved an average PCE of 17.97 ± 0.71%, while the corresponding ITO stack labeled Arc2 w ITO showed a comparable PCE of 17.57 ± 0.26%. The corresponding IPCE spectra are shown in Figure S6b. It is imperative from the results that all the ITO-processed devices in various architectures demonstrate nearly similar efficiencies when compared to their reference devices owing to insignificant losses due to sputtering damage. Moreover, we demonstrated bifacial and semitransparent PSCs with architecture identical to Arc2 (labeled trans). When illuminated from the glass side, the semitransparent devices also demonstrated a superior PCE of 15.4 ± 0.51%. A detailed summary of the different architectures is found in Table 1. In addition, minimodules with an architecture Arc2 were fabricated. Figure 5b illustrates the comparison of *J*–*V* characteristics of ITO-based PSCs and modules with an area of 0.09 and 2.31 cm^2^, respectively. The PCE of 18.24% for the cell and 16.42% for the module is in line with the results obtained for simple architectures.

**Table 1:** Summary of the Photovoltaic Parameters of Various PSC Architectures w and w/o ITO Sputtering at Optimized Sputtering Parameters

variation	*V*_oc_ (V)	*J*_sc_ (mA cm^2^)	FF (%)	PCE (%)	*R*_s_ (Ohm cm^2^)	*R*_p_ (Ohm cm^2^)
ITO/MeO-2PACz/PFN-Br/Cs_0.05_MA_0.14_FA_0.81_PbI_2.7_Br_0.3_-IL-Oam-BHC/PCBM/SnO_x_/Cu (**Arc1 w/o ITO**)	1.06 ± 0.03 (1.09)	21.69 ± 0.39 (22.26)	80.42 ± 1.63 (82.25)	18.54 ± 0.69 (19.39)	5.58 ± 0.69 (4.58)	18357.27 ± 30055.69 (8865.0)
ITO/MeO-2PACz/PFN-Br/Cs_0.05_MA_0.14_FA_0.81_PbI_2.7_Br_0.3_-IL-Oam-BHC/PCBM/SnO_x_/ITO/Cu (**Arc1 w ITO**)	1.06 ± 0.01 (1.08)	22.±0.25 (22.31)	78.96 ± 1.71 (80.76)	18.44 ± 0.55 (19.05)	6.89 ± 4.03 (5.43)	1765.88 ± 4047.68 (15030.0)
ITO/PTAA/PFN-Br/Cs_0.05_MA_0.14_FA_0.81_PbI_2.7_Br_0.3_-IL-OAm-BHC/PCBM/SnO_x_/Cu (**Arc2 w/o ITO**)	1.08 ± 0.02 (1.09)	21.84 ± 0.72 (22.74)	76.5 ± 0.85 (77.45)	17.97 ± 0.71 (18.76)	6.15 ± 0.03 (5.66)	908.7 ± 248. (301.5)
ITO/PTAA/PFN-Br/Cs_0.05_MA_0.14_FA_0.81_PbI_2.7_Br_0.3_-IL-Oam-BHC/PCBM/SnO_x_/ITO/Cu (**Arc2 w ITO**)	1.06 ± 0.003 (1.06)	21.35 ± 0.25 (21.6)	77.87 ± 0.05 (77.92)	17.57 ± 0.26 (17.82)	5.79 ± 0.44 (6.46)	2003.96 ± 3321.6 (4482.0)
ITO/PTAA/PFN-Br/Cs_0.05_MA_0.14_FA_0.81_PbI_2.7_Br_0.3_-IL-Oam-BHC/PCBM/SnO_x_/ITO (**Trans**-glass-side)	1.06 ± 0.01 (1.07)	20.05 ± 0.76 (21.05)	72.67 ± 4.06 (77.39)	15.4 ± 0.51 (15.92)	6.23 ± 0.61 (5.33)	229.94 ± 79.42 (353.6)
ITO/PTAA/PFN-Br/Cs_0.05_MA_0.14_FA_0.81_PbI_2.7_Br_0.3_-IL-Oam-BHC/PCBM/SnO_x_/ITO (**Trans-**ITO side)	1.03 ± 0.02 (1.05)	19.15 ± 0.55 (20.21)	72.05 ± 3.49 (76.14)	14.27 ± 0.79 (14.95)	7.1 ± 0.5 (6.5)	6978.4 ± 13388.62 (3312.0)

## 3 Conclusions

In summary, we have analyzed how the sputtering parameters affect the performance of the PSCs in the inverted architecture. Our experiments reveal that UV/plasma also contributes significantly to the overall damage induced by the sputtering process. PL data suggests that despite the presence of the SnO_x_ buffer layer, sputtering damage due to UV/plasma is evident. By optimizing the ITO thickness, we demonstrate that at least 100 nm is needed to inhibit the metal migration. In addition, high RF power densities of the sputtering yield an additional penalty of about 30% in the cell. By carefully optimizing the deposition parameters, we reduced the damage and achieved performances identical to the reference. We demonstrated excellent photovoltaic performance with a PCE of 18.44 ± 0.55%, which is on par with the reference device with a PCE of 18.54 ± 0.69%. Superior light-soaking stability with a *T*_80_ of 1426 h and an impressive thermal stability of 1530 h, retaining 94.5% of initial efficiency, were observed. Moreover, to demonstrate the effectiveness of the optimization, we realized minimodules with significant improvements in the PCE up to 16.42% and thermal stability with a *T*_80_ of 350 h. Overall, this work establishes robust sputtering protocols and paves the way for efficient and stable single-junction, semitransparent devices, and tandem applications.

## 4 Experimental Section

### 4.1 Materials

Dimethylformamide (DMF-anhydrous-Sigma-Aldrich), dimethyl sulfide (DMSO-anhydrous-Sigma-Aldrich), toluene (anhydrous-Sigma-Aldrich), chlorobenzene (CB-anhydrous-Sigma-Aldrich), dichlorobenzene (DCB-anhydrous-Sigma-Aldrich), poly[bis(4-phenyl)(2,4,6-trimethylphenyl)amine] (PTAA) (Solaris Chem), MeO-2PACz (TCI), PFN-Br (Sigma-Aldrich), [6,6]-phenyl-C61-butyric acid methyl ester (PCBM-99%-Solenne), bathocuproine (BCP-96%-Sigma-Aldrich), SnO_x_ ink (N30-Avantama), methylammonium bromide (MABr-99.99%-Greatcell solar), formamidinium iodide (FAI-99.99%-Greatcell solar), cesium iodide (CsI-99.99%-Sigma-Aldrich), lead bromide (PbBr2-TCI), lead iodide (PbI2-TCI), 1-butyl-3-methylimidazolium tetrafluoroborate (BMITFB-98%-ACROS), oleylamine (OAm-Sigma-Aldrich), and benzylhydrazine hydrochloride (BHC-Life Chemicals) were purchased and used without further purification. Glass/ITO substrates (10 Ω/sq) were purchased from Kin-Tec.

### 4.2 Device Fabrication

A UV nanosecond laser was used to etch the ITO/glass substrates (10 Ω/sq, 2.5 × 2.5 cm^2^). The patterned substrates were cleaned in an ultrasonic bath using a detergent solution (2% Hellmanex in deionized water), subsequently with deionized water, followed by acetone and 2-propanol, 15 min for each step. After drying, they were treated for 15 min in a UV/O_3_ tool (Novasonic). PTAA (2 mg/mL) dissolved in toluene was spun at 5000 rpm for 30 s and annealed for 10 min at 100 °C. After cooling down, the samples were treated under UV light for 5 min to wet the surface of PTAA. MeO-2PACz (0.33 mg/mL) dissolved in ethanol was spun at 4000 rpm for 30 s and annealed for 10 min at 100 °C. For the device architecture where PFN-Br is used, the substrates were spun with a solution (0.5 mg/mL of DMF) at 5000 rpm for 30 s, followed by annealing the substrate to 100 °C for 10 min. The perovskite precursor was spin-coated at 4000 rpm for 35 s; subsequently, 180 μL of CB was dropped after 20 s. The film was annealed for 10 min at 100 °C. PCBM was spun at 1700 rpm for 30 s and annealed at 100 °C for 5 min. BCP was spun at 4000 rpm. SnO_x_ nanoparticle dispersions (purchased from Avantama AG, 2.5 wt % suspension in a mixture of butanol) were filtered with a PVDF 0.45 μm filter and spun at 5000 rpm for 35 s; subsequently, the films were annealed at 120 °C for 20 min. After that, the devices were sputtered with ITO in the Kenosistic (KS400) sputtering machine with a tin-doped indium oxide (304.8 mm × 76.2 mm 90% In_2_O_3_–10% SnO_2_) target using a low-temperature RF magnetron sputtering with a base pressure of 1.2 × 10^–6^ mbar and a working pressure of 1.1 μbar, a power density of 0.18 W cm^–2^, and a precursor argon flow at 40 sccm unless specified otherwise. High-quality ITO films were grown at room temperature in an oxygen-free environment. The temperature of the carrier was found to be 40 °C for a power density of 0.344 W cm^–2^. The samples were placed on a pallet and moved parallel to the target at a speed of 120 cm/min. The thickness was optimized by changing the number of scans of the pallet. The sheet resistance of the as-deposited sputtered ITO layer was 30 Ω/sq for a thickness of 100 nm. Finally, the 100 nm copper electrode was deposited by thermal evaporation. The active area of this electrode was fixed at 0.16 cm^2^ using a shadow mask.

### 4.3 Module Fabrication

P1 ablation was performed on ITO-coated glasses (10 Ω/sq, 2.5 × 2.5 cm^2^) using a UV 365 nm laser beam. Figure S5a depicts the schematic of the module layout. The module consists of three series-connected cells with 0.76 cm^2^ active per cell, accounting for an overall active area of 2.28 cm^2^. The module geometrical FF, that is, the ratio between the active area and the aperture area, is 90%. After the P1 laser ablation, the patterned substrate was cleaned in an ultrasonic bath, using a detergent with deionized water, acetone, and isopropanol (15 min for each step). The cleaned ITO glass (2.5 cm × 2.5 cm) was deposited with PTAA, PFN-Br, perovskite, PC_61_BM, BCP, and SnO_x_ using the method mentioned for the fabrication of cells. P2 ablation was then performed to remove the device stack on the interconnection areas. The laser parameters were optimized to remove PTAA/perovskite/PCBM/SnO_x_ stack on modules. The samples were charged in a high-vacuum chamber (1 × 10^–6^ mbar) to sputter ITO. 100 nm-thick ITO was sputtered at a working pressure of 1.1 μbar, a power density of 0.344 W cm^–2^, and a rate of 1.04 nm/min by scanning the substrates parallel to the target. After the deposition, the samples were thermally evaporated with Cu (nominal thickness 100 nm). Finally, P3 ablation was performed using the same laser system, and parameters were optimized to remove the ITO/Cu contacts to obtain the electrical insulation between the top electrodes of adjacent cells.

### 4.4 Characterizations and Measurements

The morphologies of ITO (0, 10, 40, and 100 nm) on the SnO_x_ substrate were examined using field emission SEM (FESEM, Tescan Mira 3 LMU FEG) and an optical microscope (Olympus, OLS3100). The optical microscopic pictures were obtained from the light incident side. The absorption spectra were measured in the wavelength range of 300 to 900 nm using a UV–vis spectrophotometer (Shimadzu UV 2550). The photovoltaic characteristics of the devices were observed using a class A solar simulator (ABET) under AM 1.5G 1 Sun illumination conditions. The AM1.5G condition is obtained by using an optical filter. The sun simulator was calibrated using a Si reference cell (RR-226-O, RERA Solutions). The *J–V* curves were measured in both forward and reverse directions from 0.1 to 1.2 V and vice versa. The step voltage and dwell duration were both set to 20 mV and 50 ms. By masking the active region, the *J*–*V* curves of all the devices were measured in ambient air (25 °C, 30% RH). Electroluminescence of the modules was measured with a homemade setup with raspberry-pi camera v2.1 containing an IMX219 sensor. The EL images for modules were with an exposure of 5000 ms under a voltage of 3.5 V and 2.7 mA.

### 4.5 Long-Term Stability Tests

For long-term stability tests, PSC devices were protected with Kapton tape without any robust encapsulation method. ISOS-L1 was performed by MPPT using an Arkeo-multichannel (Cicci Research) based on 32 totally independent Source Meter Units (±10 V@±250 mA) and an ARKEO Light soaker (VIS version) with a low mismatch LED-based system (400–750 nm) at 0.7 sun. With a *J–V* scan every 2 h, a conventional Perturb & Observe tracking method was chosen. ISOS-D-2 was carried out in an oven maintained at 85 °C. The PCE of the device was periodically measured using a class A solar simulator (ABET) with a xenon lamp under AM1.5G simulated sunlight (1 sun) after cooling the device down to room temperature for over 2 h. A comparison of the LED light and xenon lamp spectra is provided in Figure S7.

## Supporting Information Available

Effect of aging of ITO devices on performance statistics, spectrum of UV–plasma, effect of SnO_x_ on different electrodes, ITO sheet resistance at different power densities and working pressures, ITO transmittance spectra, electroluminescence data of minimodules, IPCE, and comparison of different sun-simulator spectra (PDF)
