# Supplementary material for: Holistic Approach toward a Damage-Less Sputtered Indium Tin Oxide Barrier Layer for High-Stability Inverted Perovskite Solar Cells and Modules
Source: ACS Appl Mater Interfaces. 2022 Nov 2;14(45):51438–48. doi: 10.1021/acsami.2c10251 (PMC9673066; doi:10.1021/acsami.2c10251)
Supplement: Supplementary file 1 — am2c10251_si_001.pdf [file am2c10251_si_001.pdf]

# Supporting Information

## Holistic Approach toward a Damage-Less Sputtered ITO Barrier Layer for High Stability Inverted Perovskite Solar Cells and Modules

Sathy Harshavardhan Reddy[1], Francesco Di Giacomo[1], Fabio Matteocci[1], Luigi Angelo Castriotta[1] and Aldo Di Carlo[1,2]*

[1]Centre for Hybrid and Organic Solar Energy (CHOSE), University of Rome Tor Vergata, Rome 00133, Italy;

[2]ISM-CNR, Institute of Structure of Matter, National Research Council, Rome 00133, Italy;

orcid.org/ 0000-0001-6828-2380 Email: aldo.dicarlo@uniroma2.it

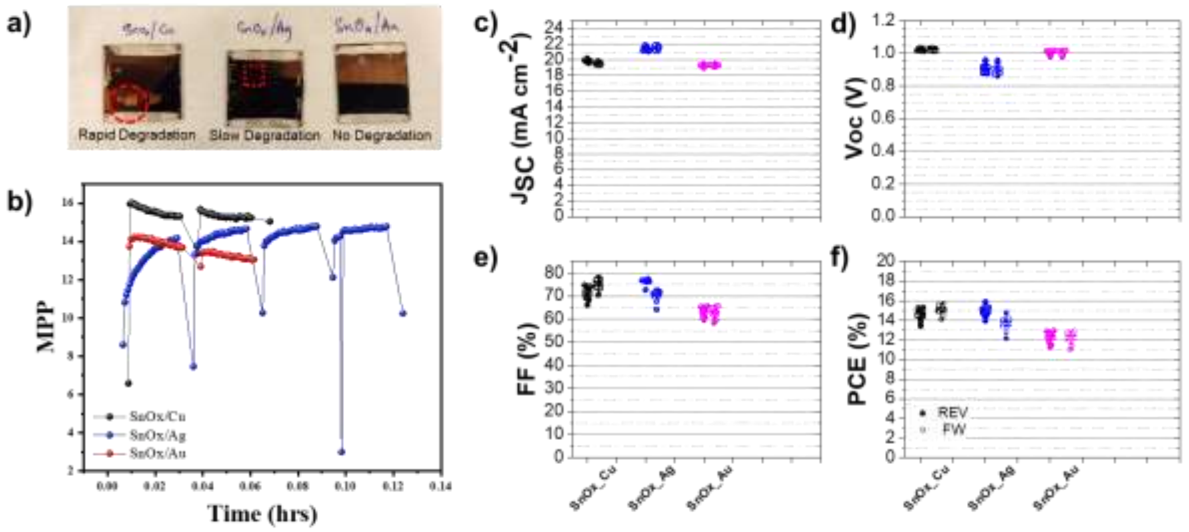


**Figure S1.** Effect of $SnO_x$ on different a) Image showing the degradation of $SnO_x$-based PSC with different electrodes (Cu (left), Ag(middle), Au (right)) when encapsulated and stored under ambient conditions. b) MPPT tracking of the solution-processed low-stable $SnO_x$ based devices with different electrodes. Device performance for perovskite solar cells with different electrodes. (c) $J_{sc}$, (d) $V_{oc}$, (e) FF, and (f) PCE. Solid filled symbol and open symbol indicate the JV parameters measured under the reverse-bias (REV) and forward-bias (FW) scan directions,

respectively.

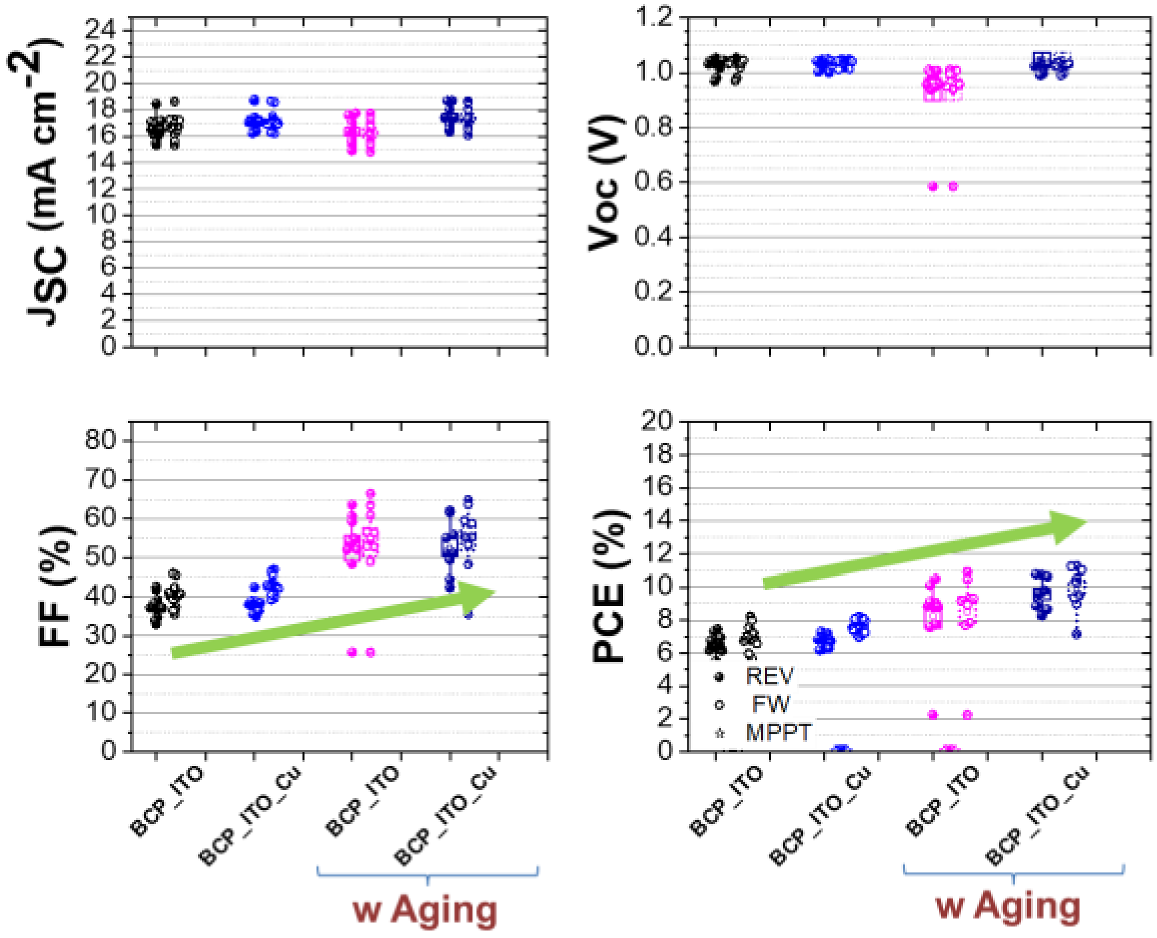


**Figure S2.** Performance statistics of BCP/ITO/Cu devices before and after aging for 2 weeks. Solid filled symbol and open symbol  indicate the JV parameters measured under the reverse-bias (REV) and forward-bias (FW) scan directions, respectively.

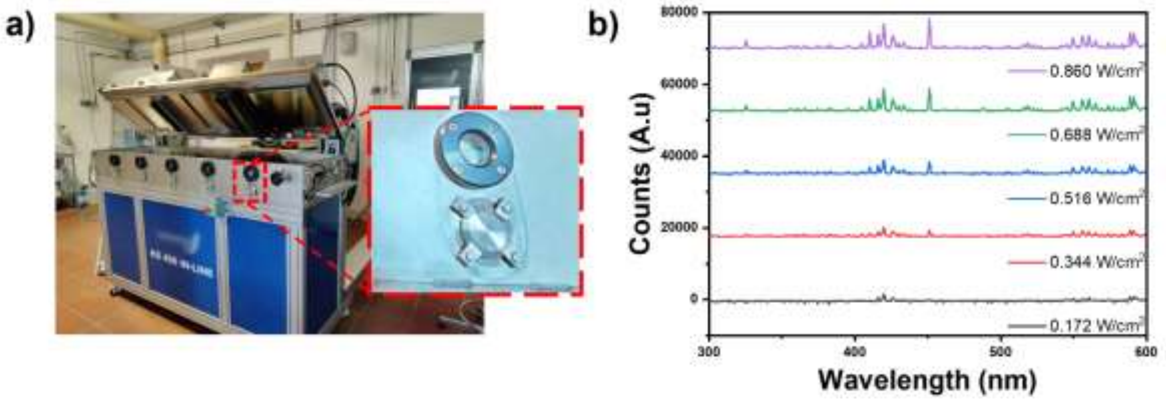


**Figure S3.** (a) Image of sputtering setup showing the window from which the spectrum is measured (b) Spectrum measured by Spectrophotometer

| Variation | Voc V | Jsc mA cm² | FF % | PCE % | Rs Ohm | Rp Ohm |
|---|---|---|---|---|---|---|
| **ITO 0nm** | 1.±0.01 (1.02) | 19.55±1.17 (21.28) | 68.38±4.4 (74.) | 13.45±1.61 (15.7) | 7.67±1.01 (6.66) | 511.98±516.8 (1539) |
| **ITO-10nm** | 1.04±0.01 (1.06) | 21.5±0.37 (21.87) | 65.14±4.94 (70.07) | 14.6±1.05 (15.64) | 7.42±0.11 (7.34) | 529.2±605.85 (1386) |
| **ITO-40nm** | 1.05±0.02 (1.06) | 21.58±0.31 (22.15) | 59.16±3.2 (62.71) | 13.42±0.9 (14.42) | 9.66±0.78 (8.98) | 667.53±426.42 (1566) |
| **ITO-100nm** | 1.02±0.02 (1.04) | 19.99±0.66 (21.27) | 48.72±1.85 (51.04) | 9.95±0.41 (10.67) | 16.17±0.72 (15.45) | 608.67±683.37 (2124) |

Table S1. Summary of the main photovoltaic parameters of PSCs with different thicknesses at a power density 0.258 W $cm^{-2}$ and a working pressure of 1.1 μbar measured initially.

| Variation | Voc V | Jsc mA cm² | FF % | PCE % | Rs Ohm | Rp Ohm |
|---|---|---|---|---|---|---|
| **No ITO** | 0.94±0.05 (1.01) | 14.94±1.87 (18.07) | 47.62±6.64 (57.64) | 6.61±0.81 (8.27) | 89.11±66.59 (17.82) | 689.34±685.72 (2916) |

| | | | | | | |
|---|---|---|---|---|---|---|
| **ITO-10nm** | 1.00±0.03<br>(1.02) | 18.98±1.12<br>(20.42) | 60.01±5.13<br>(66.38) | 11.47±1.69<br>(13.64) | 10.62±4.08<br>(7.49) | 1116.±1292.17<br>(4059) |
| **ITO-40nm** | 1.03±0.02<br>(1.05) | 19.42±0.57<br>(20.02) | 56.89±8.32<br>(65.59) | 11.32±1.6<br>(12.92) | 9.91±2.03<br>(7.48) | 305.82±246.21<br>(764) |
| **ITO-100nm** | 1.03±0.01<br>(1.04) | 22.72±0.72<br>(23.63) | 66.22±4.05<br>(69.31) | 15.42±1.03<br>(16.5) | 7.32±2.19<br>(6.05) | 983.25±1164.33<br>(5013) |

Table S2. Summary of the main photovoltaic parameters of PSCs with different thicknesses at a power density 0.258 W cm$^{-2}$ and a working pressure of 1.1μbar measured after 2 weeks.

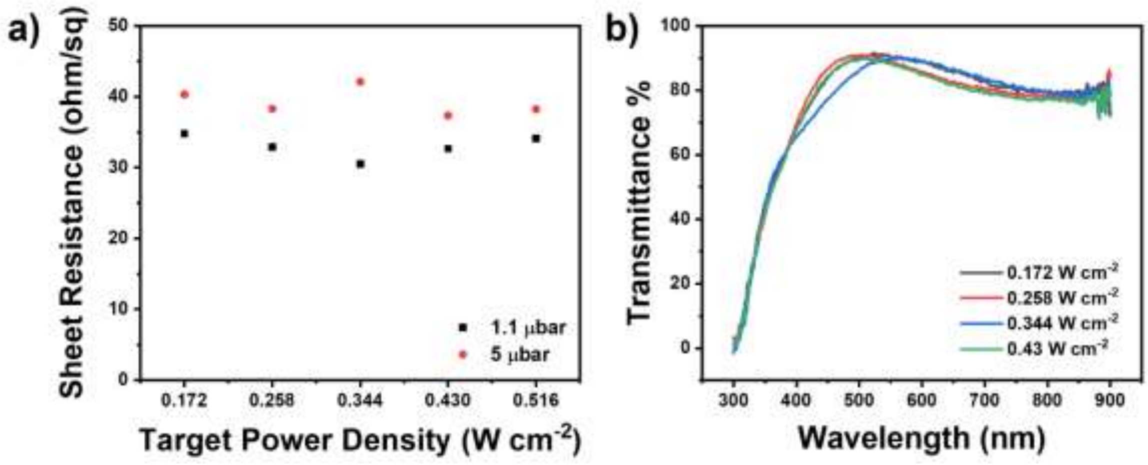


**Figure S4** (a) Sheet resistance of ITO sputtered at different powers and working pressures. (b) Transmittance spectra of ITO films deposited at various target powers and a working pressure of 1.1 μbar.

| **Variation** | **Voc**<br>**V** | **Jsc**<br>**mA cm$^2$** | **FF**<br>**%** | **PCE**<br>**%** | **Rs**<br>**Ohm cm$^2$** | **Rp**<br>**Ohm cm$^2$** |
|---|---|---|---|---|---|---|
| **Cu** | 0.94±0.02<br>(0.97) | 15.1±1.3<br>(17.99) | 48.47±4.59<br>(58.49) | 6.89±1.05<br>(9.13) | 21.9±3.9<br>(16.2) | 537.7±625.2<br>(1107) |
| **40W**<br>**(**0.172 W cm$^{-2}$**)** | 1.05±0.01<br>(1.07) | 21.4±0.7<br>(22.27) | 70.61±2.59<br>(73.65) | 15.95±0.92<br>(16.78) | 6.6±0.6<br>(6.0) | 611.4±572.9<br>(1674) |
| **60W**<br>(0.258 W cm$^{-2}$) | 1.05±0.02<br>(1.07) | 20.8±0.7<br>(21.91) | 67.39±2.82<br>(73.25) | 14.74±0.83<br>(15.93) | 7.7±1.0<br>(7.1) | 495.7±906.9<br>(441.9) |

| | | | | | | |
|---|---|---|---|---|---|---|
| **80W** (0.344 W cm$^{-2}$) | 1.03±0.02 (1.05) | 22.1±0.4 (22.82) | 67.2±4.08 (71.67) | 15.37±0.96 (16.35) | 8.3±2.1 (5.9) | 324.4±96.3 (477.9) |
| **100W** (0.43 W cm$^{-2}$) | 1.05±0.01 (1.06) | 20.4±0.3 (21.07) | 64.2±2.26 (67.46) | 13.79±0.59 (14.4) | 8.3±0.6 (8.3) | 800.5±746.5 (281.7) |
| **120W** (0.516 W cm$^{-2}$) | 1.00±0.04 (1.06) | 20.3±1.0 (21.83) | 54.87±5.71 (63.71) | 11.18±1.48 (13.44) | 11.5±2.5 (10.0) | 420.7.±403.6 (275.4) |

Table S3. Summary of the main photovoltaic parameters of PSCs with ITO sputtering at different power densities at a working pressure of 1.1 μbar.

| **Variation** | **Voc** V | **Jsc** mA cm$^{2}$ | **FF** % | **PCE** % | **Rs** Ohm cm$^{2}$ | **Rp** Ohm cm$^{2}$ |
|---|---|---|---|---|---|---|
| **40W** (0.172 W cm$^{-2}$) | 1.03±0.02 (1.03) | 21.0±0.5 (21.96) | 61.43±4.7 (68.22) | 13.27±1.28 (15.25) | 9.04±2.05 (6.6) | 1364.3±1672.6 (237.6) |
| **60W** (0.258 W cm$^{-2}$) | 1.02±0.02 (1.04) | 21.2±0.9 (22.44) | 62.47±5.41 (66.81) | 13.41±1.02 (14.84) | 7.8±0.76 (7.0) | 503.7±434.4 (1161) |
| **80W** (0.344 W cm$^{-2}$) | 1.02±0.02 (1.04) | 20.9±1.4 (22.82) | 62.04±4.77 (71.45) | 13.25±1.45 (15.24) | 9.88 ±1.54 (9.5) | 2095.3±4103 (338.4) |
| **100W** (0.43 W cm$^{-2}$) | 1.01±0.06 (1.04) | 20.4±1.2 (21.96) | 51.61±5.9 (58.79) | 10.64±1.72 (13.32) | 12.9±2.03 (11.8) | 296.1.±128.5 (262.8) |

Table S4. Summary of the main photovoltaic parameters of PSCs with ITO sputtering at different power densities at a working pressure of 5 μbar.

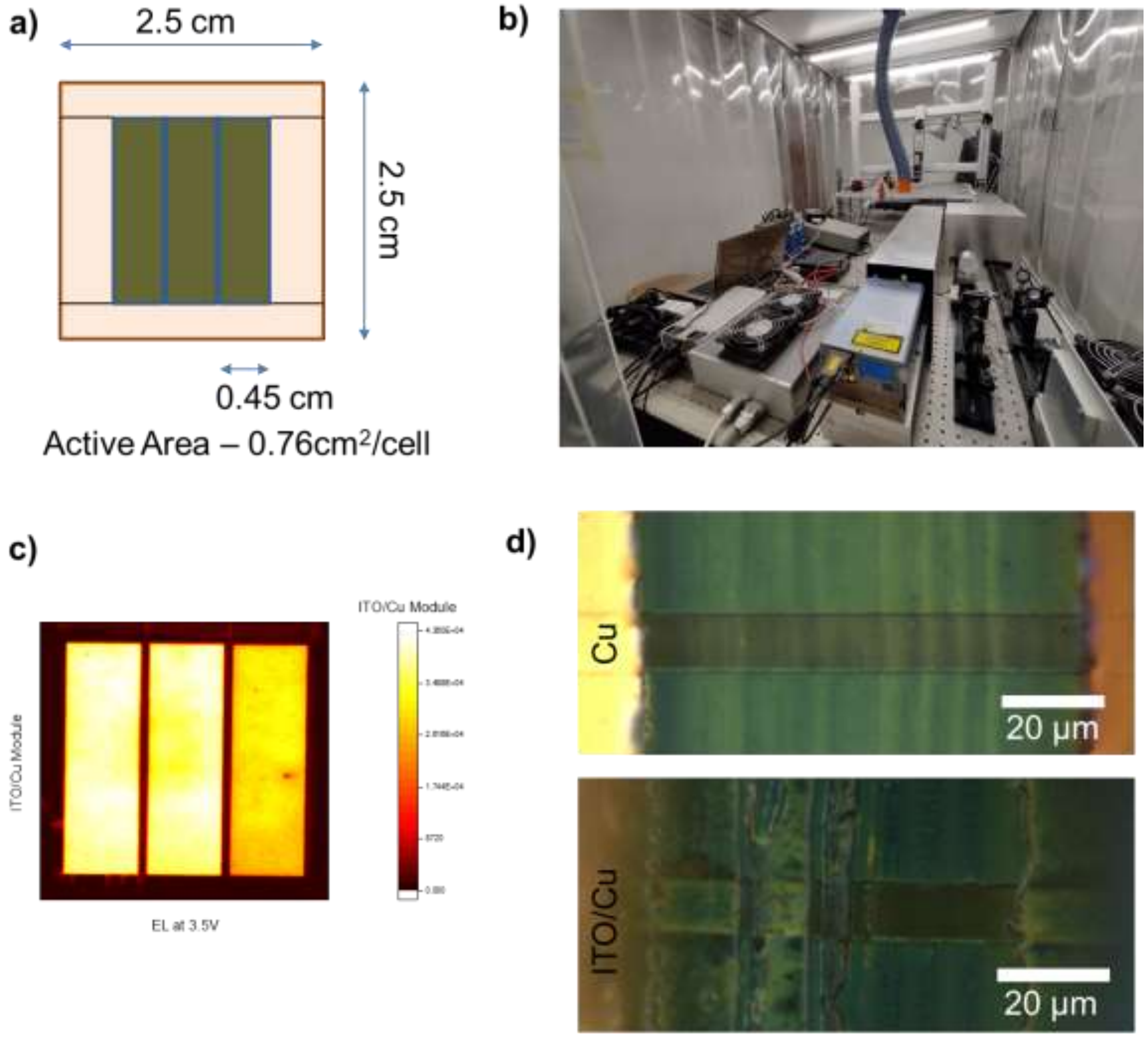


**Figure S5.** (a) Scheme of mini module layout (b) Picture of Laser setup room (c) Electroluminescence images of ITO/PTAA/CsFAMA-IL/PCBM/BCP/$SnO_x$/ITO/Cu mini-module illustrating the defects in the perovskite layer. (d) Top showing the optical microscope image of complete P3 ablation of Cu electrode. Bottom showing the optical microscope image of leftover debris of P3 ablation of ITO/Cu electrode on a mini-module.

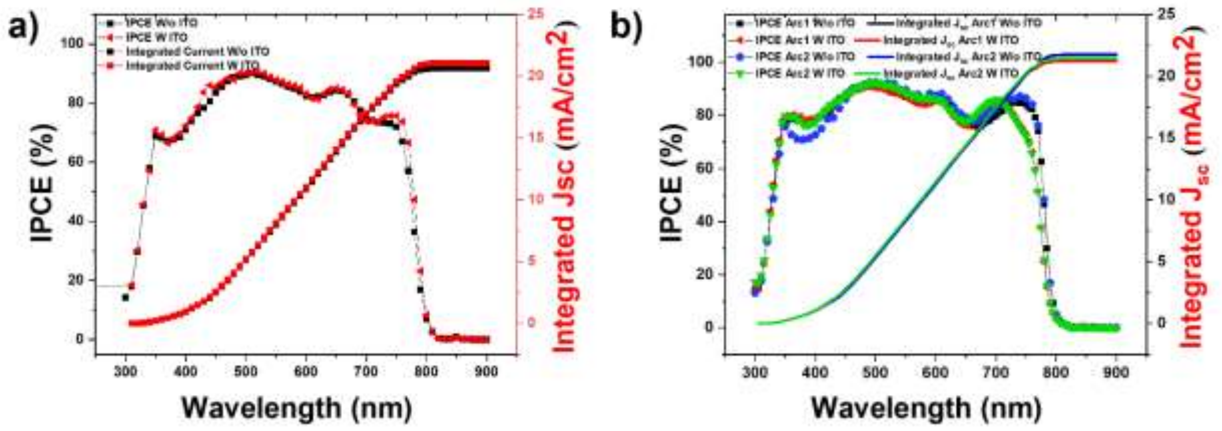


**Figure S6**: Comparison of IPCE spectra with and without ITO for different architectures. a) simple architecture b) advanced architectures

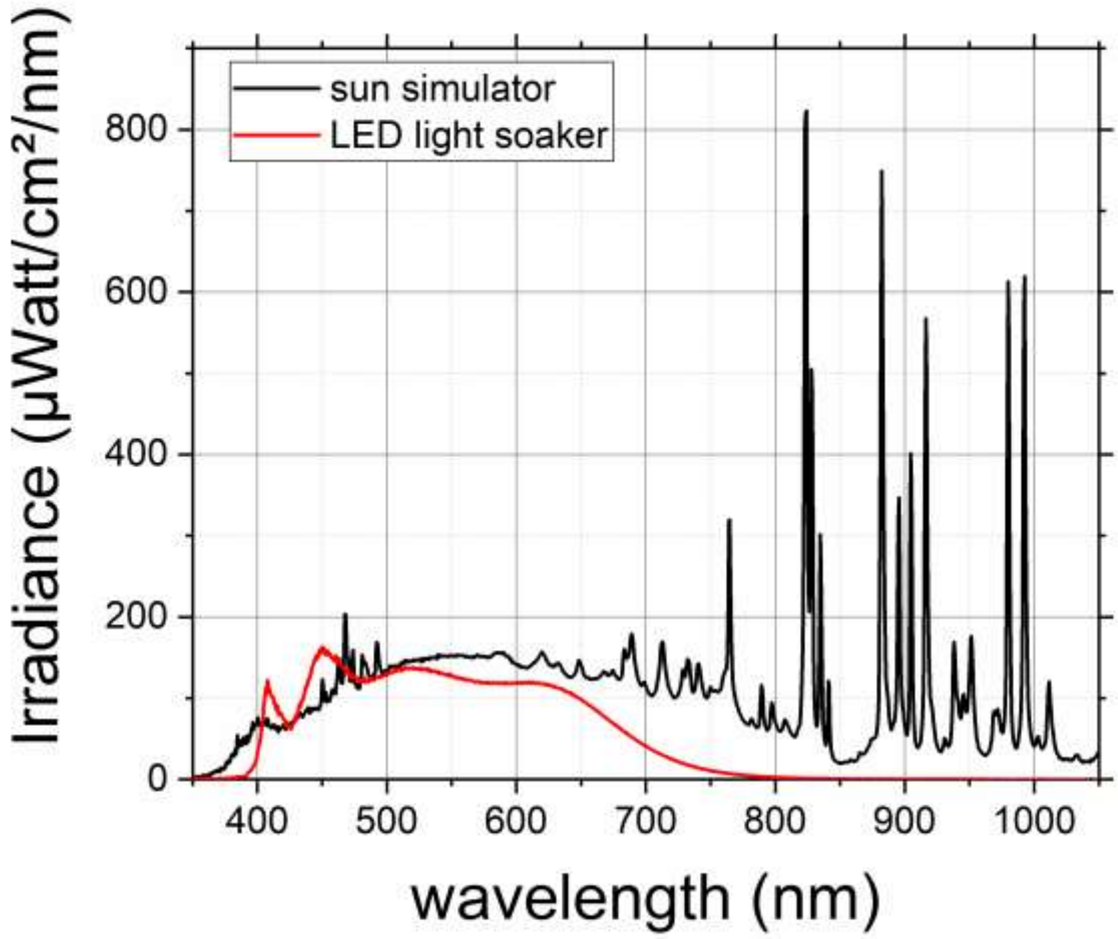


**Figure S7**: Comparison of spectrum of the sun-simulator and LED light soaker.
